# Supplementary material for: The Characterization and Evaluation of the Soluble Triggering Receptor Expressed on Myeloid Cells-like Transcript-1 in Stable Coronary Artery Disease
Source: Int J Mol Sci. 2023 Sep 4;24(17):13632. doi: 10.3390/ijms241713632 (PMC10487797; doi:10.3390/ijms241713632)
Supplement: Supplementary file 1 [file ijms-24-13632-s001.zip › ijms-2572342-supplementary.pdf]

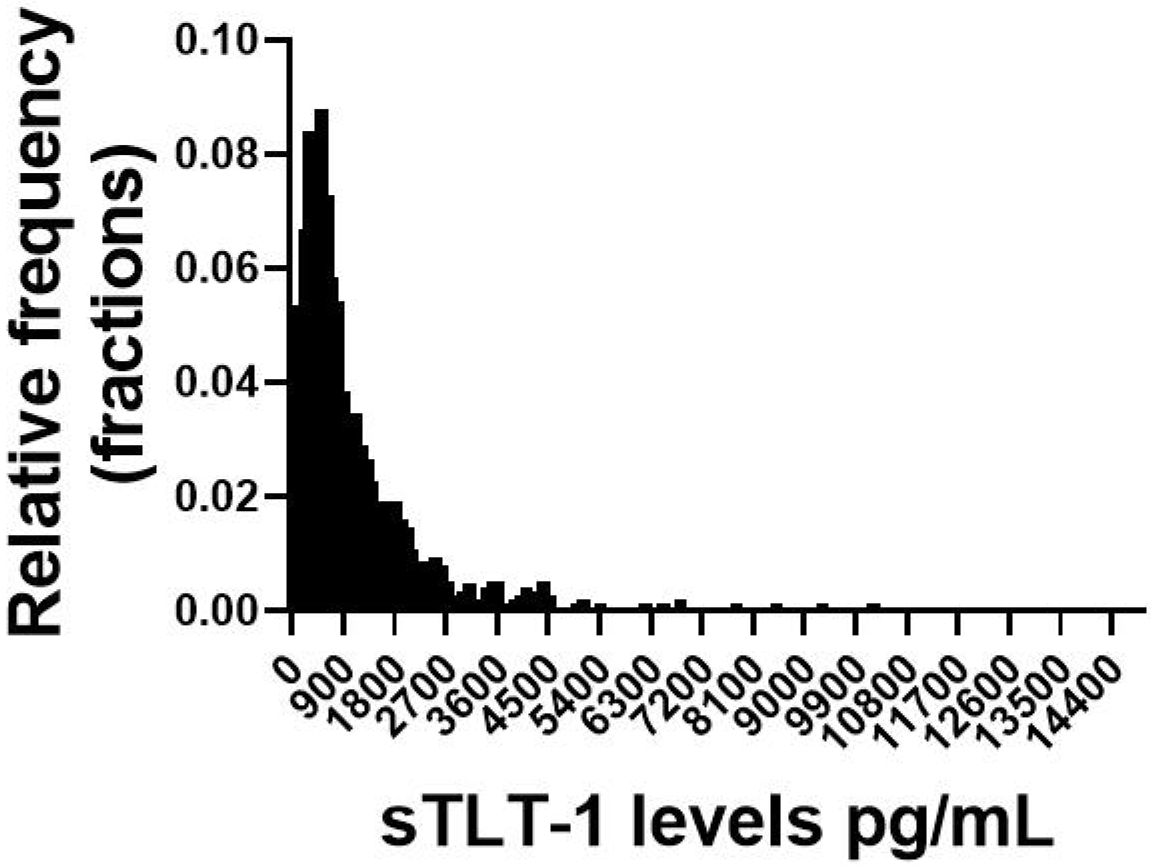

Supplemental figure 1 - sTLT-1 Frequency of distribution

sTLT-1 Frequency of distribution from patients in the PEACE clinical trial. The distribution figure was completed with a bin size of 100 pg/mL (Prism 9). The peak distribution was completed with a 5 pg/mL bin size.
